# The nexus between digitalization and sustainability: A scientometrics analysis

**DOI:** 10.1016/j.heliyon.2023.e15172

**Published:** 2023-04-14

**Authors:** Leila Irajifar, Hengcai Chen, Azadeh Lak, Ayyoob Sharifi, Ali Cheshmehzangi

**Affiliations:** a School of Architecture & Urban Design, RMIT University, Australia; b Department of Architecture and Built Environment, University of Nottingham Ningbo China, Ningbo, China; c Faculty of Architecture and Urban Planning, Shahid Beheshti University of Tehran, Tehran, Iran; d Network for Education and Research on Peace and Sustainability (NERPS), Hiroshima University, Hiroshima, Japan; e Graduate School of Humanities and Social Sciences, Hiroshima University, Japan

**Keywords:** Digitalization, Sustainability, Smart cities, Sustainable development goals, Scientometric analysis

## Abstract

Digitalization and sustainability are among the most critical mega-trends in 21st century. The nexus between digitalization and sustainability unfolds exciting opportunities in addressing global challenges, creating a just and sustainable society and laying the groundwork for achieving the Sustainable Development Goals. Several studies have reviewed the link between these two paradigms and how they mutually impact one another. However, most of these reviews are qualitative and manual literature reviews that are prone to subjectivity and so lacking the required rigor. Given the above, this study aims to provide a comprehensive and objective review of the knowledge base on how digitalization and sustainability actually and potentially contribute to each other and highlight the key research that links these two megatrends. A comprehensive bibliometric analysis of academic literature is conducted to objectively visualize the research status quo across time, disciplines, and countries. The Web of Science (WOS) database was searched for relevant publications published between January 1, 1900, and October 31, 2021. The search returned 8629 publications, of which 3405 were identified as primary documents pertaining to the study presented below. The Scientometrics analysis identified prominent authors, nations, organizations, prevalent research issues and examined how they have evolved chronologically. The critical review of results reveals four main domains in research on the nexus of sustainability and digitalization including Governance, Energy, Innovation, and Systems. The concept of Governance is developed within the Planning and Policy-making themes. Energy relates to the themes of emission, consumption, and production. Innovation has associated with the themes of business, strategy, and values & environment. Finally, systems interconnect with networks, industry 4.0, and the supply chain. The findings are intended to inform and stimulate more research and policy-making debate on the potential interconnection between sustainability and digitization, particularly in the post-COVID-19 era.

## Highlights

• 3405 journal articles were analyzed through the combinations of Scientometrics analysis and critical literature review.
• Major scientific collaboration networks in sustainability and digitalization research are visualized and analyzed.
• Keywords co-occurrence and evolution of research in the intersection of digitalization and sustainability was visualized.
• Four research themes and the corresponding challenges digitalization for sustainability were identified.

## 1 Introduction

The 21st century is seen as the ‘century of planetary survival’. The rapid population growth combined with vigorous industrialization, urbanization and globalization push planetary boundaries and trigger more frequent and intensified disasters, loss of biodiversity, destruction of natural ecosystems, regional and social disparities etc. [1]. The ever-increasing global risks, environmental deterioration and overwhelming inequality challenges in our societies call for a radical shift in the way our societies operate, survive, and thrive. To address these issues, the concept of ‘sustainability’ has gained more interest since publication of the Brundtland Report in 1987 [2] and it has gained even further momentum since publication of the Sustainable Development Goals (SDGs) in 2015, the New Urban Agenda in 2017 and other sustainability in policy frameworks such as COP26 [3].

Alongside with climate change and planetary survival issues, the digital revolution has proved to be a key transformational force in societal changes in this century. Many scholars believe that the underlying notion of sustainable urban development is closely aligned with the concept of the digitalization process, which encourages interactions between humans and technologies for a sustainable living environment [[4], [5], [6]]. Digitalization -also called third industrial revolution-refers to the advancement of technology from analog electronic and mechanical devices to the digital technologies. It originated from the invention of transiters in 1947 which led to creation and use of computers and cellphones in 1980s and eventually led to introduction of World Wide Web in 1992. By 2000s, digital revolution spread all over the developing world and with rapid changes to technologies, industries, and societal patterns, it took another leap to the fourth industrial revolution in the last decade (2010s) with more focus on interconnectivity, automation, machine learning and real-time data [7].

Digitalization has provided new opportunities and challenges in addressing sustainability issues. Moreover, policymakers are engaged in an ongoing search for an optimal strategy to address economic, social, and ecological crises, including stagnation and growing inequality following the 2007–08 crash and rapidly accelerating climate change and environmental instability. In this context, the growth of the digital economy, innovations and technologies have emerged as a means to address these crises [8]. Therefore, bridging disciplinary perspectives or even empirical domains will become crucial as digitization and sustainable development become increasingly entwined.

The interconnection between sustainability and digitalization is considered as a winning combination (Brenner & Hartl, 2021 [9,10]; and recent studies increasingly investigates the possibility of a positive as well as a negative relationship between digitization and sustainability (Andersson & Lozano, 2021; [9,11,12]. The literature on the link between digitalization and sustainability are divided in different groups.

1) some have just focused on a particular form of digitalization (Brenner & Hartl, 2021 [9]; Isensee et al., 2020), for instance Del Rio's work is more focused on ICT [9], Beier and Reis investigated the link between Industry 4.0 and SDGs [13,14], Dantas' work is in the intersection of Circular Economy (CE) and sustainability [15], Vinuesa and Bibri have published research on the nexus of AI/big data and sustainability [16]: [17].
2) The other group in the literature investigated the varied dimensions of sustainability (e.g., ecological, economic, social) in the era of digitalization (Brenner & Hartl, 2021 [9,18]; to find the gaps in SDG research [9],
3) There are also systematic reviews just focused on the smart cities' contribution to urban sustainability (Ramirez Lopez & Grijalba Castro, 2021; Wu et al., 2022).

Given the above, this research aims to make a comprehensive and objective review of the research in the intersection of these two megatrends, and fill the gaps in the fragmented literature on the nexus of digitalization and sustainability.

This study is a bibliometric and Scientometrics analysis of the literature in the intersection of sustainability and digitalization and it seeks to identify the major research trends and emerging concepts in this area. Concerning the relationship between digitization and sustainability and a better understanding of how this research topic has evolved chronologically. Accordingly, this study can be bibliometrically analyzed in two ways. The first one is performance analysis which involves measuring scientific impact and citations via other indexes. This analysis method allows for an understanding of the research field as well as its structure, evolution, and trends in academic activity [3]. The second method is science mapping which makes possible the representation of scientific research along with its development in the intellectual and social fields [19]. Thus, we performed a bibliometric analysis of the relationship between “digitization and sustainability” using the information collected from the Web of Science (WoS) network through VOS viewer. The data sources and bibliometric methods used are described in the following section. Section 3 presents and discusses the results. Finally, the main conclusions and recommendations for further research are provided in Section 4.

## 2 Methods

This study employs a systematic review method combined with bibliometric analysis and content analysis [20,21]. These methods can track the evolving knowledge base and hot topics at the nexus between sustainability and digitization. Covering all relevant publications in a specific field of study would be challenging, especially sinceit is technologically, conceptually, politically, and scientifically immature to harness the transformative potential of sustainability and digitization [12]. Historically, scholars have conceptualized the fields of sustainability and digitalization under several collective phrases [22]. Therefore, some systematic literature analysis methods such as iterative query restatement can cover as many publications as possible through certain retrieval formulas [23]. In the present study, we implemented a similar query formulation method to search for sustainability and digitalization literature in the well-known Web of Science (WoS) Core Collection. WoS is chosen as the main database since it is one of the most important sources of citation data, and its multidisciplinary coverage is a huge advantage for comparing different scientific topics [24]. The bibliometric metadata data was retrieved from the Web of Science Core Collection database, including Science Citation Index Expanded (SCI-EXPANDED), Social Sciences Citation Index (SSCI), Arts & Humanities Citation Index (A&HCI), Emerging Sources Citation Index (ESCI) for related literature.

**Fig. 1:**
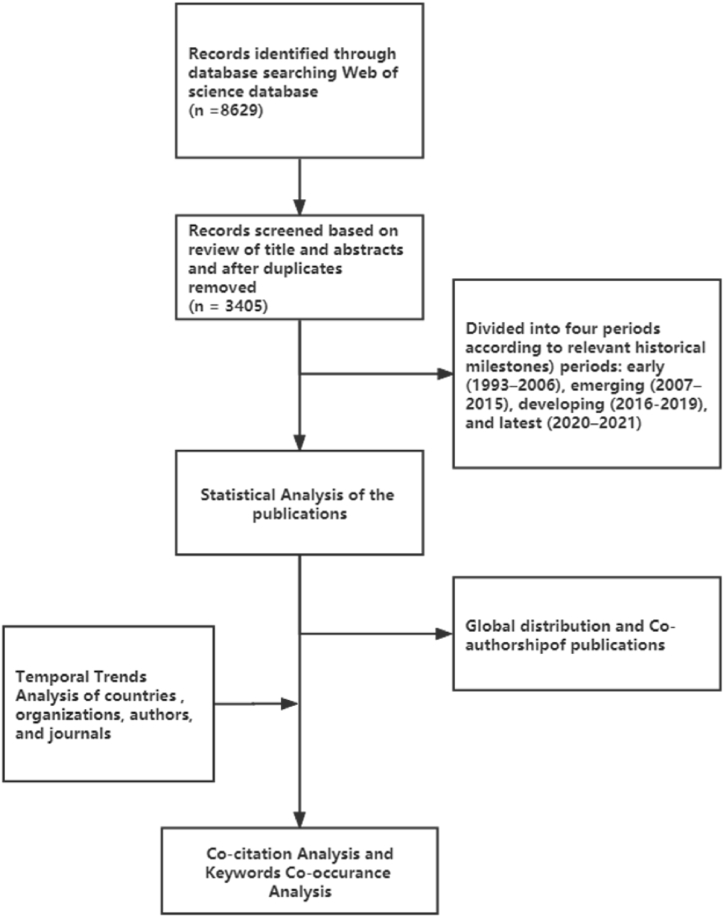
Bibliometric analysis framework.

In this study, analyses were conducted to identify and categorize the most influential journals, publications, authors, subject areas (Web of Science Core Collection Categories), temporal and spatial trends in the research (Fig. 1). Moreover, the spatial analysis conducted in two ways: (a) by author affiliation and (b) by content analysis of the manuscript text to determine the geographic focus. To analyze as many relevant publications as possible, a broad-based search string was designed that is composed of combinations of different terms related to sustainability and digitalization (see the Appendix). We only searched for publications written in English and the timespan was 1900–2021. The initial search in Titles, Abstracts, and Keywords of documents indexed in the Web of Science returned 8629 publications in October 2021.

To obtain more accurate analysis results, titles, and abstracts of all the retrieved publications were examined. Those publications that are not focused on digitalization and sustainability were excluded during the screening process, and 3405 publications were obtained for the bibliometric analysis. The sample size is suitable for text mining using VOSviewer and allows understanding the structure of the field and its trends. These cover several common types of documents such as articles, book reviews, editorial material, reviews, and so on (see Table 1). They cover several common types of documents such as articles (86.6%), reviews (11.2%), proceeding papers (1.8%), editorial material (1.8%), Book Chapters (0.3%), Book Reviews (0.3%), and others. The total frequency statistics used by document types is greater than the number of documents retrieved because documents can be divided into two different types.

**Table 1:** Overview of publications on digitalization and sustainability from Web of Science split into three time periods: early (1993–2006), emerging (2007–2015), developing (2016–2019), and latest (2020–2021).

Categories	1993–2006		2007–2015		2016–2019		2020–2021		Total	
	N	%	N	%	N	%	N	%	N	%
**Publications**	95	2.8	519	15.2	1271	37.3	1520	44.6	3405	100
**Document Types**										
Article	85	89.5	469	90.4	1561	85.7	1281	84.3	2949	86.6
Review	6	6.3	29	5.6	215	11.8	222	14.605	381	11.2
Book Review	3	3.2	6	1.2	1	0.1	0	0.0	9	0.3
Book Chapter	0	0.0	5	1.0	3	0.2	1	0.1	9	0.3
Proceedings Paper	21	22.1	14	2.7	42	2.3	1	0.1	62	1.8
**Research Areas**										
Environmental Sciences Ecology	23	24.2	86	16.6	435	34.2	625	41.1	1169	34.3
Science Technology Other Topics	4	4.2	50	9.6	406	31.9	573	37.7	1033	30.3
Engineering	21	22.1	117	22.5	260	20.5	340	22.4	738	21.7
Computer Science	25	26.3	118	22.7	169	13.3	180	11.8	492	14.4
Energy Fuels	1	1.1	35	6.7	107	8.4	134	8.8	277	8.1
Business Economics	22	23.2	86	16.6	152	12.0	194	12.8	454	13.3
Information Science Library Science	13	13.7	57	11.0	55	4.3	34	2.2	159	4.7
Public Administration	11	11.6	15	2.9	51	4.0	53	3.5	130	3.8
Telecommunications	1	1.1	38	7.3	58	4.6	52	3.4	149	4.4
Construction Building Technology	7	7.4	9	1.7	43	3.4	71	4.7	130	3.8
**Source Titles**										
Sustainability	0	0.0	17	3.3	206	16.2	315	23.1	574	16.9
Journal of Cleaner Production	1	1.1	3	0.6	57	4.5	82	5.4	143	4.2
Sustainable Cites and Society	0	0.0	0	0.0	29	2.3	48	3.2	77	2.3
Sensors	0	0.0	6	1.2	6	0.5	11	0.7	23	0.7
Renewable & Sustainable Energy Reviews	1	1.1	5	1.0	21	1.7	5	0.3	32	0.9
Energies	0	0.0	2	0.4	19	1.5	41	2.7	62	1.8
IEEE Access	0	0.0	2	0.4	19	1.5	27	1.8	48	1.4
Cities	1	0.0	1	0.2	17	1.3	7	0.5	26	0.8
Remote Sensing	0	0.0	0	0.0	10	0.8	10	0.7	20	0.6
Technological Forecasting and Social Change	4	4.2	2	0.4	17	1.3	15	1.0	38	1.1
International Journal of Sustainable Development and World Ecology	1	1.1	3	0.8	3	0.2	2	0.1	9	0.3
Automation in Construction	2	2.1	1	0.3	3	0.2	1	0.1	8	0.2
**Country or Region**										
USA	21	22.1	136	26.2	190	14.9	156	10.3	503	14.8
UK	18	18.9	53	10.2	139	10.9	159	10.5	369	10.8
Australia	8	8.4	35	6.7	67	5.3	94	6.2	204	6.0
Canada	8	8.4	26	5.0	49	3.9	52	3.4	135	4.0
Germany	6	6.3	30	5.8	59	4.6	75	4.9	170	5.0
Italy	6	6.3	39	7.5	99	7.8	126	8.3	270	7.9
China	6	6.3	49	9.4	195	15.3	288	18.9	538	15.8
**Organizations**										
Chinese Academy of Sciences	2	2.1	7	1.9	31	2.4	26	1.7	66	1.9
University of California System	1	1.1	11	2.1	12	0.9	6	0.4	30	0.9
Queensland University of Technology Qut	1	1.1	4	0.8	14	1.1	11	0.7	30	0.9
University of London	2	2.1	11	2.1	14	1.1	12	0.8	39	1.1
Royal Institute of Technology	2	2.1	5	1.0	12	0.9	7	0.5	26	0.8
University of Manchester	1	1.1	3	0.6	9	0.7	4	0.3	17	0.5
**Citations**										
Sum of Times Cited	4212	6.4	21,848	33.4	31,021	47.4	8389	12.8	65,470	100.0
Highest cited publication	1006		533		1259		154		1259	
Average Citations per item	43.34		42.1		24.41		5.52		19.23	
h-index	27		71		82		38		109	

As one of the purposes of this study is to examine the evolution of the field over time, four periods according to three relevant historical milestones (early research 1993–2006, emerging research 2007–2015, the third research 2016–2019, and the latest research 2020–2021) were identified. The first milestone is the invention of the World Wide Web in 1993 [25] which a had major impact on the digitalization process. Next is the release of the first iPhone in 2007 [26], which introduced the mass adoption of smartphones. The third milestone was 2015, when the Fourth Industrial Revolution, or Industry 4.0 began [27]. It is also the year when major international policy frameworks related to sustainability, such as the Sustainable Development Goals (SDGs) were introduced [28]. Finally, the last period starts from 2020 as we also wanted to explore impacts of the COVID-19 pandemic on this field. Other periods could have been identified, especially before 2015. However, the number of publications until then is, relatively limited not warranting additional sub-periods (i,e, a relatively large number of publications is needed for bibliometric analysis).

VOSviewer software (this Java application is available at: https://www.vosviewer.com/) is one of the common tools widely used for knowledge mapping and bibliometrics analysis [29]. The research utilizes VOSviewer to visualize and map the literature and identify dominant research topics, themes (term co-occurrence analysis), and co-author and co-citation interconnections [30,31]. As some terms are synonyms and to avoid separate counting, before the analysis, a thesaurus file was designed and introduced to the software. This ensures avoiding separate counting of synonyms such as Internet of Things and IoT.

In addition to detailed numerical information for performance analysis, the main outputs of the VOSviewer analyses are networks of nodes and links. Depending on the type of analysis, nodes refer to components such as keywords, authors, reference, and journals. Node size is proportional to the frequency of co-occurrence. For instance, in the term co-occurrence analysis, terms that have co-occurred more frequently with other terms are shown using relatively larger nodes. The width of links connecting two nodes is proportional to the strength of connection between those nodes. In other words, nodes that are strongly connected and have co-occurred more are connected using thicker links.

Overall, three major types of analyses were conducted in VOSviewer. The term co-occurrence analysis was used to identify research areas, research hotspots, and the evolution of related research. The co-citation analyses were conducted to identify most influential authors, journals, and references. Also, bibliographic coupling was done to identify countries and institutions that have made more contributions to the development of the field. For scientific details regarding these analyses, we refer readers to the VOSviewer manual (https://www.vosviewer.com/). More details about these analyses and their meanings are presented in the next section.

## 3 Results and discussions

### 3.1 Core subject areas, journals, and authors

A statistical analysis of the publications in a particular field can help us evaluate research development trends in a longitudinal process of different timelines.A preliminary search found that publications in the core collection database of the Web of Science (WoS) emerged after the 1990s. 3405 publications mainly contained Articles (N = 2949; 86.6%), Reviews (N = 381; 11.2%), and other types of documents that are focused on both digitization and sustainability based on the terms used by authors in titles, abstracts, and keywords (Table 1). Based on the Web of Science categories, the main research areas (according to the *Research Areas* in the record field exported by WoS) were ‘*Environmental Sciences Ecology*’ (N = 1169, 34.3%), ‘*Science Technology &Other Topics’* (N = 1033 30.3%), ‘*Engineering*’ (N = 738 21.7%), and ‘*Computer Science*’ (N = 492 14.4%). The journals that have contributed the most to this field are *Sustainability* (N = 574, 16.9%) and *Journal of Cleaner Production* (N = 143, 4.2%). Authors in the USA (N = 444, 15.9%), China (N = 422, 15.1%) and the UK (N = 290, 10.4%) published the most.

As shown in Table 1, the following three stages are (1) the germination period, (2) the growth period, and (3) the rapid development period. According to the database, related publications began as early as 1994. In addition, sustainability in the natural environment, society, and economic performance has always been an important research topic [32]. Since the 1990s, there has been a significant increase in the investments and potential applications of digital technology such as Information technology (IT), Information and Communication Technology (ICT) in sustainable socio-economic development [33]; Suriya and Raheem, 1998). There were very few articles on relevant studies, especially before 2000, when the annual publication was below 10. The followings are summary of the publication trends in the three indicated periods.

In the initial period (1993–2006), there were 95 documents, most of which were articles in the USA or the UK. These explorations laid the foundation for the subsequent development of digital technologies and sustainability. Most of them focused on environmental sciences, computer science, and information systems. For example, ICT impacts environmental sustainability on various levels, such as electronic waste streams, energy a product-to-service shift leading to a material-intensive economy [34]. IT also contributed to the global improvement of crop and livestock production [35]. The database shows a gradual progress in research related to the nexus between digitalization and sustainability.

The subsequent growth period (2007–2015) indicates the following: Although there were few papers during the last period, the number of publications increased year by year. Hence, there are approximately more than 50 papers per year. With the rapid development of the digital industry, the research areas in this stage range from environmental sciences, and computer science to energy, fuels, management, engineering, etc. The sustainable development of smart cities is expected to be supported by many new paradigms such as the Internet of Things (IoT) [36], 3D printing [37], machine learning [38], building information modeling (BIM) [39]. The database indicates a significant increase in related publications in the area, including the emergence of new topics that correlate between digitalization and sustainability research.

The rapid development period (2016–2019) indicates the following: After 2015, the accumulation of publications increased rapidly, and the number doubled compared to the last period. As seen from the variation trend of the number of documents, with the development of new technologies, the number of countries, journals, and authors present an increasing trend year by year, indicating that the attention level of this field shows a rising trend year by year. From 2020 to now, there is a rapid increase in the literature and scholarly research, which is partly affected by the ongoing COVID-19 pandemic.

### 3.2 An overview of the authorship and the global distribution of authors in the research area

**Fig. 2:**
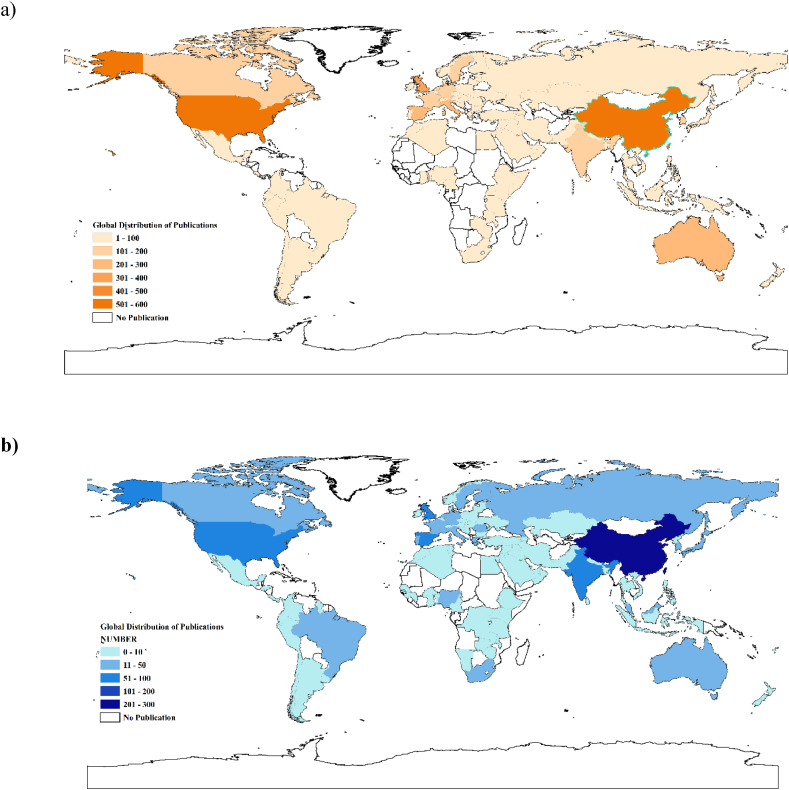
Global distribution of publications by a) the affiliation of authors and b) the country where the focus of the research was based (titles, keywords, and abstracts).

The authors draw a map based on the global distribution of authors' addresses available in WOS. This map illustrates the origin countries of the authors in this research area. To highlight the location of the cases used in these studies, the authors counted the frequency of countries or cities appearing in the titles, keywords, and abstracts of publications. For consistency in data analysis and to avoid duplication, towns or countries that appear several times in the same record are counted only once. Statistical results show that most authors are from China (N = 538;**15**.8%), the USA (N = 503;**14**.8%), and the UK (N = 369; 10.8%) (Fig. 2a), but the research sites of publications are mainly concentrated in China, the USA, India, the UK, Spain, Italy, and other countries (Fig. 2b).

**Fig. 3:**
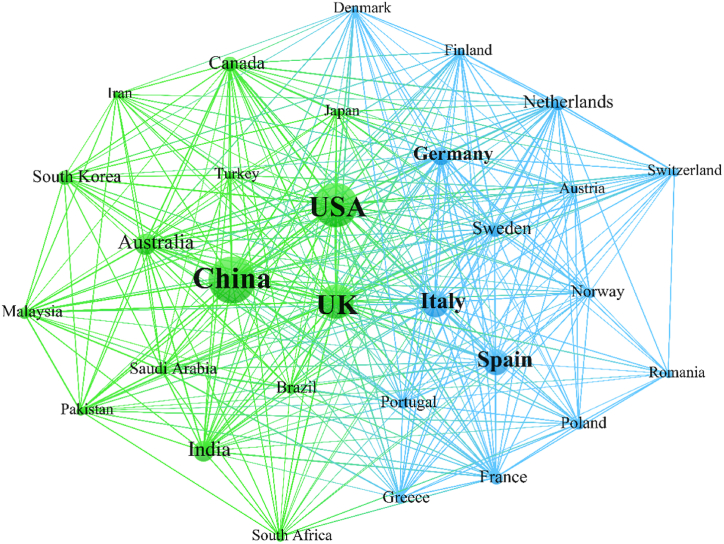
Co-authorship of 50 leading countries with at least 500 publications using VOSviewer software. Lines are weighted by the number of links, with the minimum line strength indicating two co-authored publications—the number of citations weights circles.

Two collaborative clusters emerged distinctively when analyzing co-authorship links among leading countries with at least 50 publications and 500 citations via VOSviewer software (Fig. 3). For example, the first cluster contains China (538 publications, 8723 citations, 396 link strength), which collaborates mainly with the USA (500 publications, 15,756 citations, 428 link strength), UK (365 publications, 7893 citations, 376 link strength). In contrast, in another grouping, Italy (269 publications, 5310 citations, 191 link strength), Spain (263 publications, 3361 citations, 127 link strength), and Germany (170 publications, 3294 citations, 195 link strength) work together. Several countries have weak link strength with others but have many publications and citations like South Korea (123 publications, 3894 citations, 62 link strength), Poland (92 publications, 666 citations, 59 link strength), and Greece (78 publications, 2107 citations, 70 link strength).

### 3.3 Temporal trends analysis

**Fig. 4:**
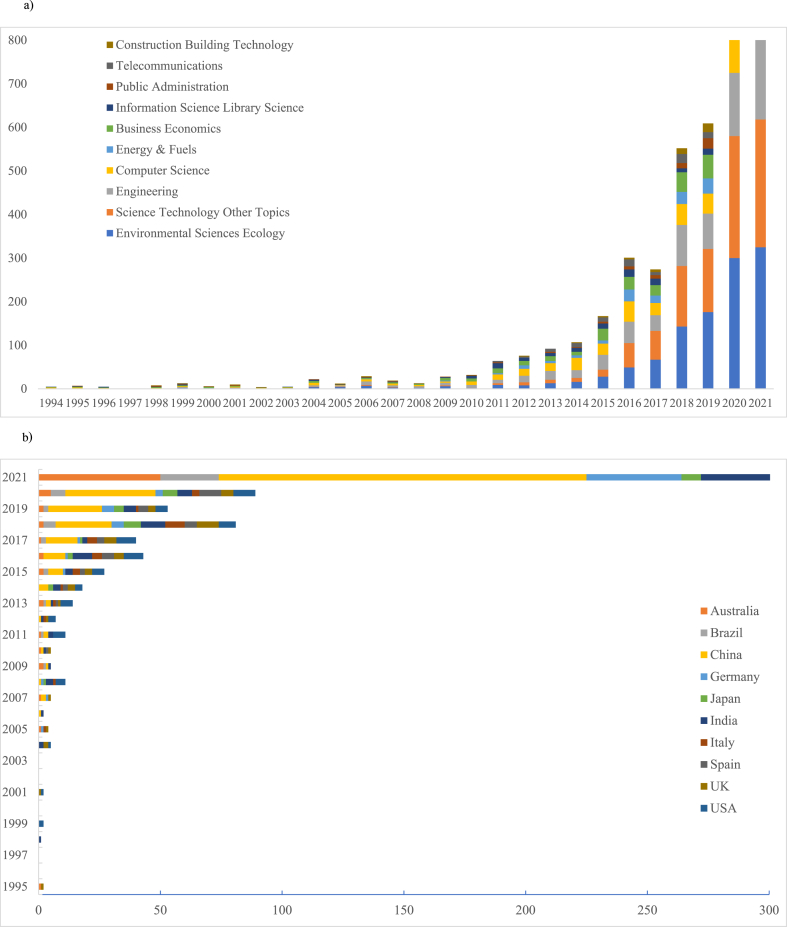

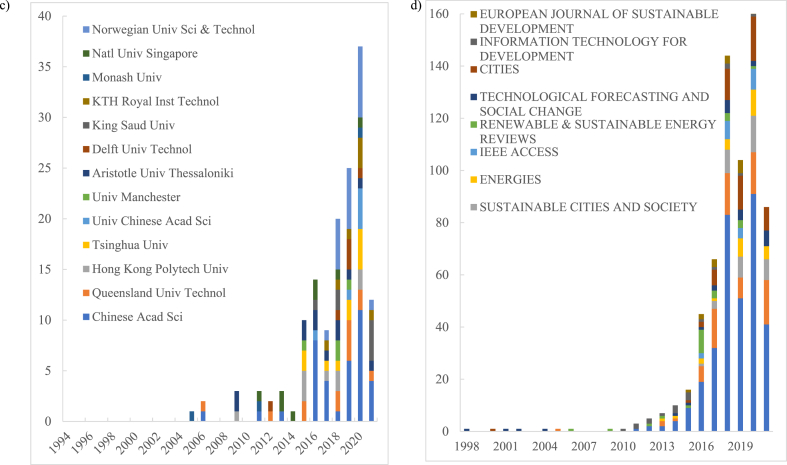
Temporal trends (1993–2021) of the cumulative number of publications by leading subject areas (a), countries based on the focus of research (b), organizations of the corresponding authors (c), and journals (d).

When we utilize Python and WOS analysis reports to split the annual records, data on four different aspects are obtained including discipline type (Fig- A), geography (Fig. 4 - B), organization (Fig. 4 - C) and research source (Fig. 4 – D). This data analysis illustrates that with the theoretical evolution, publications in this field have become more diversified over the years. It reflects in the intersection of disciplines and the participation of authors and organizations from different regions.

**Fig. 5:**
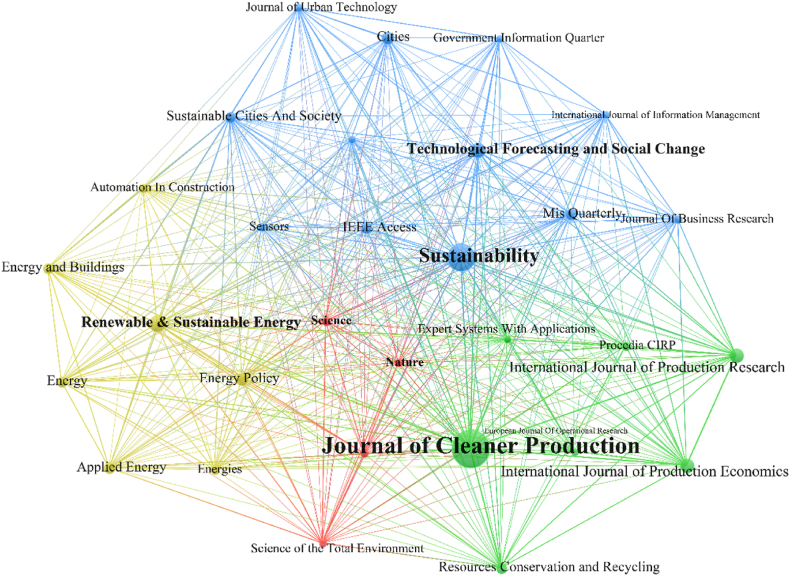
Co-citation of the most commonly cited sources. The minimum number of citations of a source is 400. The figure shows the top 30.

According to the database, the most common journals cited in the underlying literature were the Journal of Cleaner Production (5545 citations), followed by Sustainability (3448 citations), Renewable Sustainable Energy Reviews (1766 citations), and Technological Forecasting and Social Change (1482 citations) (Fig. 5).

**Fig. 6:**
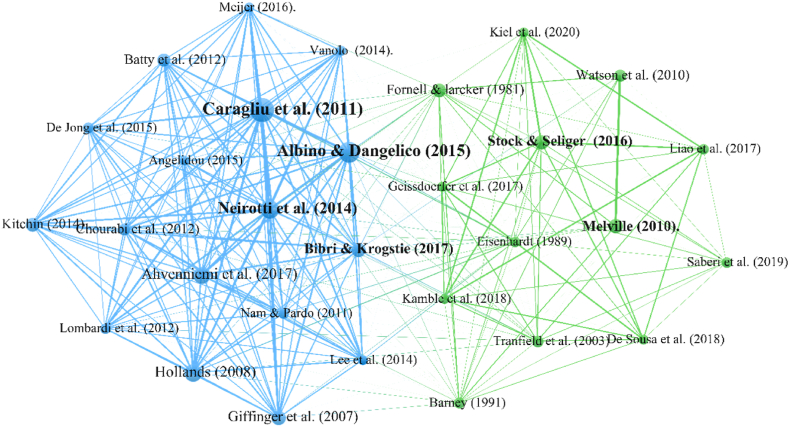
Co-citation of the most commonly cited literature. The minimum number of citations of cited literature is 50. The figure shows the top 30.

The most influential publications focus their research on topics in key areas of Smart Cities, Environment, Green, New Technology, Energy, Governance, Big data, Management, Economics. One cluster (Blue one in Fig. 6) mainly focuses on the construction sustainability and progresses of smart city. Primary studies in this cluster are [40] (136 citations, 4.0%) [41], (117 citations, 3.4%) [42], (104 citations, 3.1%) [43], (97 citations, 2.8%), Ahvenniemi (95 citations, 2.8%), and [44] (77 citations, 2.3%). For the next cluster (Green one in Fig. 8), some publications were evolved in areas like environment or green, energy. Primary studies in this clusers are [45] (78 citations, 2.3%) and [46] (63 citations, 1.9%). Some authors in this cluster mainly focus on Industry 4.0 and the circular economy, such as [47] (78 citations, 2.3%) [48], (54 citations, 1.6%) [49], (53 citations, 1.6%), de Sousa et al. (2018) (51 citations, 1.5%) [50], (51 citations, 1.5%), and [51] (51 citations, 1.5%). Other research discusses other key topics, such as big data, by Ref. [52] (73 citations, 2.1%), blockchain, by Ref. [53] (54 citations, 1.6%), and modeling, by Ref. [54] (73 citations, 2.1%).

### 3.4 Keywords Co-occurrence analysis

**Fig. 7:**
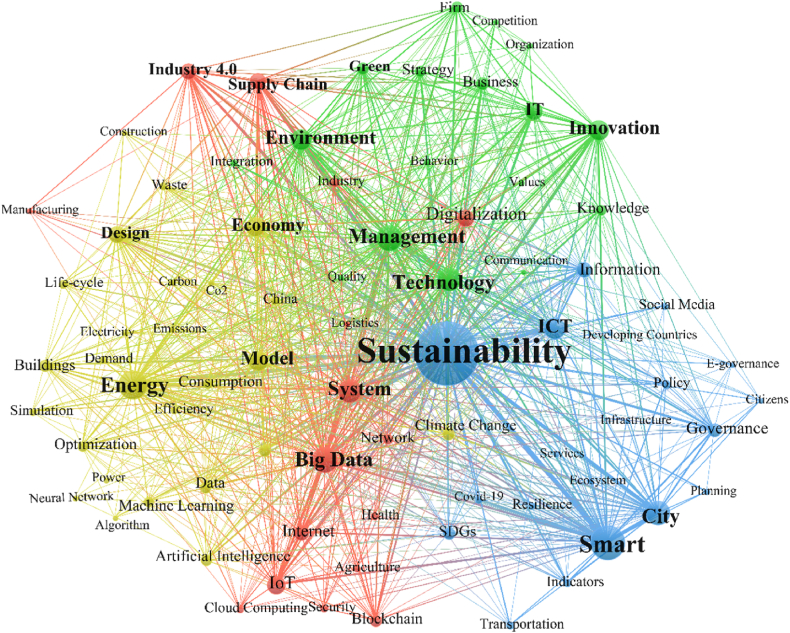
Co-occurrence of all keywords (four major themes) in the literature based on the 80 most commonly used keywords.

**The inter-related theme in the nexus of sustainability and digitiz**ation; The keywords highlight the focus of research topics and changes in trends. Keywords co-occurrence analysis is a common bibliographic analysis method to explore research fields and identify new research trends as keywords basically represent a precise summary of the research (Fang et al., 2017). In this paper, the VoSviewer tool is used to first extract the articles’ keywords, and then calculate the frequency and draw the keyword co-occurrence network to identify the frontiers of the research on sustainability and digitalization. After commonly used vocabulary and generic literature terms (*such as people, things, world, future, choice, challenges, growth, case studies, bibliographies, bibliometrics*, *literature review*, etc.) were excluded, and synonyms were merged, 12,044 keywords (including 8973 author keywords in the Vosviewer keywords mapping) were obtained. The network map of 80 keywords with a minimum of 40 co-occurrences is presented in Fig. 7 Which clearly depicts four clusters of research in the intersection of sustainability and digitalization. The blue cluster is mostly focused on Governance, Planning & Policy Making; the yellow cluster is about Energy, Emission, Consumption & Production; the green cluster is focused on Innovation, Economy, Green & Environment; and finally, the red cluster represents Systems, Networks, Industry 4.0 and Supply Chain.

**Fig. 8:**
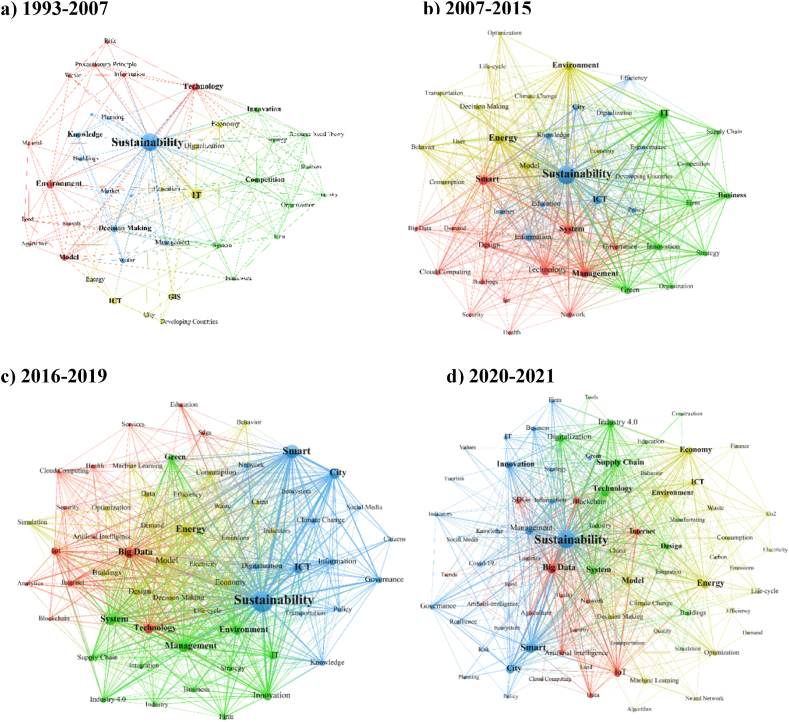
Major themes in the literature based on the most commonly used all keywords for each of the four-time periods a) 1993–2007, b) 2007–2015, c) 2016–2019, d) 2020–2021.

The keyword co-occurrence analysis was also conducted in each of the four time periods (Fig. 8). The results show that generally, with the increasing volume of publications over time, the research topics diversify with more specialized focused research. In the early period (1993–2007), the research focused on more generic themes of decision-making, innovation, competition, IT, and Environment (Fig. 8 – A). In the emerging period (2007–2015), when digitalization has taken a huge leap forward with the prominence of smart phones around the world and the introduction of industry 4.0, topics such as smart cities, systems, energy, and cloud computing (Fig. 8 – B). In the developing period (2016–2019), with the introduction of SDGs and a big push on the sustainability front, we see research on energy, big data modelling, smart cities flourish with more specialized branches in different fields such as supply chain, education, health etc (Fig. 8 – C). The focus on big data, systems, and industry 4.0 emerges in the third period and rapidly grows in the fourth period (2020–2021) when the world has been tackling a pandemic (Figs. 8–4). In this period, there is also a focus on the advancement of digitalization on e-services and supply chain demand. Research focusing on energy, management, ICT, environment, green, and technology has been primary areas throughout all periods.

#### 3.4.1 Blue cluster: governance, planning, policymaking

The combination of keywords in the blue cluster (Fig. 7) suggests a distinct theme of research that is more relevant to governance, planning, policymaking, e-governance, transportation, infrastructure, etc. The opportunities and challenges offered by disruptive dynamics of digitalization call for an urgent shift in corresponding regulatory policies, incentives, and change of perspectives, which is currently limited to a few sectors and a few countries.

Digitalization and new technologies present significant opportunities for governments, communities, and the private sector to achieve a more sustainable future. However, policymakers need to acknowledge that managing collective issues such as sustainability is at root a political, rather than a technological task. This understanding is essential for getting the best out of digitalization trends. There are great risks in bringing digital technologies into contested governance spaces if they are not shaped properly to engage the community and capacities of societies and geared to sustainability Agenda. Thus, digitalization could magnify already existing problems such as inequalities and eroding social cohesion. However, there are many ways that data and technology can be used for good if it reflects on our values with inclusive governance and democratic participation are at the heart of digital endeavors.

The other emerging theme in the blue cluster is smart and resilient cities. Smart cities are believed to be essential drivers in the sustainable and comprehensive development of society, economy, environment, resources, and risk mitigation adaptation to climate change [5]. In general, technological innovations may be applied in numerous fields ranging from enhancing energy efficiency to supporting behavioral changes and mobility patterns and governance [55]. Smart energy, smart transportation, smart buildings, smart disaster prevention and mitigation, smart municipal infrastructure, and other sustainable and smart ecological systems are being recognized in urban construction via the use of ICT and are supposed to realize the sustainable and comprehensive development of society, economy, environment, and resources [5].

The term ‘Developing Countries’ is another interesting keyword emerging in this cluster. It indicates that existing research efforts related to digitalization and sustainability in developing countries are mostly focused on enhancing compatibility, providing adequate access, sharing urban datasets, and in general, e-governance, planning and policymaking. The research in this area may provide a more localized foundation and significantly strengthen the ability to adopt new policies regarding sustainable technologies to ensure that developing countries can also become drivers of transformations toward sustainable digital societies. Smart cities have been the center of attention in developed countries like Germany, the United Kingdom, the United States, Germany, Italy, and Spain. Recently, there has been a tendency towards the fast development of smart cities in developing countries like China [5] and India [56,57]. Thus, we see faster growth of research in such contexts.

#### 3.4.2 Yellow cluster: energy, emission, Consumption & Production

One of the major themes in the nexus between digitalization and sustainability is the connection between energy, economy, emission, consumption, and production. This theme is evident in the yellow cluster in our analysis (Fig. 7) with keywords like energy, emission, electricity, demand, waste, manufacturing, etc. As energy comes mainly from fossil fuels, the exponential increase in greenhouse gas (GHG) emissions, predominantly CO2, results from the spike in global energy demands [58]. Thus, energy management is a crucial theme in this cluster as it directly relates to emissions. Also, emerging economies such as Brazil, India, China, and South Africa seek to discover reliable and affordable energy sources to fulfill their manufacturing and production needs [59], which is another key theme in this cluster.

Energy efficiency and sustainability assessment require tools and techniques based on innovative concepts such as Machine Learning, Artificial Intelligence (AI), and cloud computing. These tools and techniques can contribute to sustainability through modelling, optimization, and simulations for energy efficiency, reducing carbon emissions, life-cycle assessments, resources, and waste management. For example, the clean energy generated by a smart grid can be efficiently distributed to the society through a more stable and reasonable method of storage to allow for more effective consumption, reduction in emissions, and sustainable development of society, economy, and environment [5,59].

Machine learning (ML) is a key tool in analyzing real-world data for energy efficiency assessment in decision-making problems [60]. It has been widely used in modeling, designing, and predicting energy systems [61]. The emphasis on the vitality of Machine Learning models is because of their accuracy, robustness, precision, and generalization ability in energy systems (Donti & Kolter, 2021). Artificial intelligence (AI) is claimed to alter corporate practices and industries and address fundamental societal issues such as sustainability (Nishant, Kennedy, and Corbett, 2020). And using life-cycle assessments and circular economy principles can considerably contribute to sustainable consumption and waste management by encouraging reusing and re-manufacturing of products to improve productivity [62]. All these themes are clustered in yellow in our keyword analysis in Fig. 5 Including decarbonization and energy (energy efficiency, carbon emission, electrification, etc.) and consumption (sufficiency, pollution, waste, etc.) and production (resource use, manufacturing, circular economy, etc.) and digital tools like ML and AI.

#### 3.4.3 Green cluster: innovation, business, strategy, values & environment

Today's cities have many challenges ahead, one of which is combining competitiveness and innovation with sustainable urban development [63]. Businesses, organizations, and companies need to act quickly to embed digital transformation within their business strategy to stay relevant in today's world. The co-occurrence analysis in this study (Fig. 7) signifies this theme by clustering keywords like innovation, business, management, strategy, firm, organization, green, and environment. This combination suggests that green innovation is characterized by novelty, value, and knowledge, resulting in resource conservation and environmental improvement [64]. refer to a variety of digital innovations for sustainable development, such as green technologies that trace, control, and prevent pollution while ensuring that the entire process of product manufacture, application, and consumption has a low environmental impact [65]. Additionally, green technology could help save energy, reduce pollution, effectively manage environmental externalities, and improve ecologically [65].

Digital sustainability initiatives such as AI, ML, and big data analytics can greatly contribute to the achievement of global sustainable development goals by enabling fundamental changes in business models as well as promoting a new level of institutional accountability. The latest World Economic Forum Framework for Business Action [66], indicates that digital transformation of companies and organizations needs to be integrated with clear sustainability strategies, including their business models, supply chain, values, culture operations, and investments. Investing in digital innovations and combining them with sustainability initiatives amplifies competitiveness in companies to become tomorrow's leaders.

As a result of growing environmental consciousness, governments are developing green initiatives in light of technical innovation [67]. [68] signify the management role in sustainability by CSR management, organizational development and change, strategic management, change agents and leadership, organizational learning, sustainable organization, stakeholder engagement, sustainable business models, and business case for sustainability. Corporate social responsibility (CSR) management and corporate sustainability management have become central themes in the interlink of business, corporations, and the environment. A CSR management with a pro-active approach to environmental awareness and social well-being combined with data-led decision making will contribute to a more sustainable society by attracting better talent, receiving more capital, and providing the capability to track data and measure environmental and social impacts.

#### 3.4.4 Red cluster: systems, networks, industry 4.0 and supply chain

Industry 4.0, as the latest digital revolution trend, has been the focus of several research studies in the intersection of sustainability and digitalization, especially in our study's last two time periods (2015–2019) and (2020–2021). The red cluster in Fig. 7 represents the most recent research focus in the nexus of sustainability and digitalization, which is more related to systems and network-based solutions, including Industry 4.0, IoT, Cloud Computing, Blockchain, and Big Data in the supply chain, health, and agriculture. The interconnectivity in Industry 4.0 provides the foundation for flexible automation, cloud computing, computer simulations, cyber-physical systems, Internet of Things, collaborative and cognitive robotics, additive manufacturing, and other I.4. Technologies. Using a combination of these advanced technologies in different sectors of the economy, such as the supply chain, health, manufacturing, and agriculture, significantly influences the natural environment. Such influences result in reducing energy consumption, pollution, and greenhouse gas emissions. Simultaneously, it may contribute to an increase in profits. Industry 4.0 can reduce the environmental impact of a product, a process, or a service based on footprint data availability and traceable analysis [69]. In addition, it supports leveraging efficiency of functions such as reducing the resource consumption. Therefore, Industry 4.0 might contribute to the field of sustainability by developing sustainable digital operations to meet SDGs goals. The potential impacts of digitalization on areas like socio-environmental sustainability is still unknown, however, the increasing development of smart technologies is seen to further affect sustainability [13].

The supply chain is one of the main areas that digitalization can contribute significantly to sustainability. Common examples are by adopting a multidimensional (the environmental, economic, and social impact) and multi-scale approach (institutional, geographical, and temporal) [69]. Innovative technologies in Industry 4.0 can improve the efficiency and reliability of the internal operations and supply chain stakeholders creating a smart connection from all phases to enhance product development, procurement, manufacturing, logistics, suppliers, customers, and service [70]. The importance of incorporating sustainability in industry 4.0 initiatives is more evident in the latest period in our study (2020–2021). The global pandemic during this time provided unique challenges and opportunities that inevitably inflected the digitalization trend. The latest Mckenzie & Company report [71] on this topic indicated that Industry 4.0 technologies played a crucial role in the pandemic response at many companies, and those who had not employed Industry 4.0 before the COVID-19 got a wake-up call.

## 4 Conclusions on the new trends in the nexus between digitalization and sustainability

There has been vast research on different aspects of sustainability and digitalization as two main mega-trends in 21st century. However, the nexus between the two concepts is still underexplored and there is very limited knowledge of the contributions of the two concepts to each other and the nature of their linkages. In this study, a Scientometrics analysis was conducted on the relevant literature from WOS and the results identified key trends and domains as below.

### 4.1 Contributions to theoretical research

The trend analysis shows an exponential increase in the number of publications during the Covid global pandemic (2020–2021) which confirms the critical role of digital technologies in addressing pandemic issues. The trend analysis also reveals diversification in the topics until 2019 and then limited again to computer science, technology, and the environment in 2020–2021. In general, in the early period (1993–2007), there has been scattered research on decision-making, innovation, competition, IT, and environment, and the field experienced very slow growth in the emerging period (2007–2015). After introduction of SDGs in 2015, the research in this nexus has leaped forward in the developing period (2016–2019), focusing on energy, extensive data modeling, and smart cities flourishing with more specialized branches in various fields. The focus on new technologies contains big data, and industry 4.0 has rapidly grown in recent years.

The study also investigated the countries, institutions, journals, and authors that have contributed the most to this field and discussed relevant concepts, theories, and discourses. China, Australia and Germany are leading the research in this field in recent years. Journal of Cleaner Production, Sustainability and Technological Forecasting and Social Change have published the most relevant publication in the nexus of sustainability and digitalization.

The co-occurrence analysis highlighted the key terms in the intersection of digitalization and sustainability and revealed four main domains in the nexus of these two megatrends. The first domain is focused on governance, planning and policymaking and provides the essential elements for the emergence of smart and resilient cities to respond in times of crisis appropriately as well as respond to social-political. In addition, progress in this domain can lead to social innovation with social media capabilities facilitating the empowerment of a sustainable society. The second domain deals with the themes of energy, emission, consumption, and production which uses digitalization in modeling, optimization, and simulation for optimal consumption and production and economic growth. The third domain refers to the environmental challenges using innovative business models and emerging green technologies. Finally, the fourth domain is related to industry 4.0, systems and networks.

### 4.2 Contributions to practice and application

The results of this study not only could guide research institutions to identify and prioritize changing research trends, but also government or corporate decision-makers can identify and prioritize areas for development and further exploration, including in the areas of smart cities, energy, and economic development. The emerging technologies that can be applied in these areas can be obtained from the analysis of our four thematic modules. The findings can help these policy makers plan their sustainability strategies and create a virtuous circle for stakeholders. These digital technologies can trigger new business models that are environmentally friendly and can also create better profitability models through the digitization of production processes.

### 4.3 Reliability and limitations

The interplay between digitalization and sustainability offers opportunities to shape a greener economy and society. Several reviews of the literature and studies have carried out explorations of this area. For example, Chen et al. (2020) discusses the overall impact of digitization on environmental sustainability in the context of manufacturing [72]. provide a systematic overall review of the literature in the field of SDGs and digitization by providing an overview of the SDGs and their relationship to digitization. Mondejar et al. (2021) describe the opportunities that digitalization offers for building a sustainable society of the future in terms of food production, drinking water, and green energy. These existing studies are biased toward qualitative research, while the study is biased toward quantitative research. Feroz et al. (2021) outlined four key areas including pollution control, waste management, sustainable production, and urban sustainability. Lertpiromsuk and Ueasangkomsate (2022) used bibliometric analysis in the Scopus database to classify regions for important keywords in three areas: sustainable business innovation, sustainable manufacturing development, and sustainable digital economy. The conclusions reached in these studies have some commonalities with the present study, thus confirming the reliability of the findings. Current research and practice are still in the early stages, and the authors suggest further exploration of research and policy implications. The sample of this study was relatively large, so during the validation of the data, in addition to the automatic statistics based on the software and database, the authors also manually calibrated and checked the results through programming and other means to ensure the reliability of the results.

The methods and procedures conducted in this research were not without limitations. For instance, the time division in this study is based on the distribution of important events subjectively selected and the number of documents obtained. These preconditions of data collation may lead to some deficiencies in the analysis. Besides, the selection of keywords may not be comprehensive enough with the increasing number of papers. Therefore, we need to investigate further the overall trend and development of a research branch or topic to broaden the research horizon. To facilitate the interpretation of the data, the authors identified only the English literature and this unique database. In addition, we acknowledge that bibliometric analysis should be complemented with systematic literature reviews to gain a more detailed understanding of the field.

### 4.4 Future research directions

Digitalization has great potential to overcome social problems and development gaps, and sustainability is a necessary condition for a low-carbon digital transformation. Digitalization can also involve environmental impacts, social divisions, and ethical issues. Due to the complex multidimensional nature of both digitization and sustainability, further research on how this interaction generates new policy and governance approaches will be the main thrust of future research.

Overall, this study has provided a basis for further discussion of the substantive and synergistic implications of emerging and future digital technologies, particularly the forms of function, planning, and development required for future social, economic, and environmental sustainability practices. However, challenges revolve around the uncertainty, contradiction, and fallacy of the practical application and tremendous knowledge explosion. The emerging and future digitalization will also have novel applications, data analysis capabilities, and user-friendly services. Therefore, they can make a substantial contribution to social progress and quality of life - not only in facilitating the process of sustainable development but also in operating and managing development by constantly assessing and anticipating their contribution to sustainable development.

## Declarations

### Author contribution statement

Leila Irajifar: Conceived and designed the experiments; Contributed reagents, materials, analysis tools or data; Wrote the paper. Hengcai Chen: Performed the experiments; Wrote the paper. Azadeh Lak: Analyzed and interpreted the data; Contributed reagents, materials, analysis tools or data; Wrote the paper. Ayyoob Sharifi: Conceived and designed the experiments; Performed the experiments; Contributed reagents, materials, analysis tools or data. Ali Cheshmehzangi: Conceived and designed the experiments; Analyzed and interpreted the data.

### Data availability statement

No data was used for the research described in the article.

### Declaration of interest’s statement

The authors declare the following conflict of interests: Ali Cheshmehzangi reports financial support was provided by National Natural Science Foundation of China (10.13039/501100001809NSFC), and the Network for Education and Research on Peace and.Sustainability (NERPS), Hiroshima, Japan. Ali Cheshmehzangi acknowledges the National Natural Science Foundation of China (10.13039/501100001809NSFC) for the provision of funding for project number 71950410760. He also acknowledges the Ministry of Education, Culture, Sports, Science and Technology (10.13039/501100001700MEXT), Japan Government, and the Network for Education and Research on Peace and Sustainability (NERPS), Hiroshima, Japan.

## Declaration of competing interest

The authors declare the following financial interests/personal relationships which may be considered as potential competing interests:Ali Cheshmehzangi reports financial support was provided by NERPS. Ali Cheshmehzangi reports a relationship with NERPS that includes: funding grants.

## Appendix

The search string:

TS=(“sustainability” OR “sustainable development”) AND (“digitali*ation” OR“digital technolog*” OR “Information and communication technolog*” OR “ict” OR “information technolog*” OR “internet of things” OR “iot” OR “artificial intelligence” or “AI” or “machine learning” OR “blockchain” OR “virtual reality” OR “VR” OR “augmented reality” OR “AR” OR “3-d print*” OR “three-dimensional printing” OR “cloud computing” OR “big data” OR “5G” OR “6G” OR “Smart technolog*” OR “smart home*” OR “smart house*” OR “smart cit*” OR “home energy management system*” OR “industry four*” OR “society 5*” OR “robotic*” OR “automation” OR “unmanned aerial vehicle*” OR “UAV*” OR “smart meter*” OR “smart grid” OR “vehicle-to-vehicle communication” OR “machine-to-machine communication”)
